# The effect of cyclodextrin-solubilized curcuminoids on amyloid plaques in Alzheimer transgenic mice: brain uptake and metabolism after intravenous and subcutaneous injection

**DOI:** 10.1186/alzrt170

**Published:** 2013-03-28

**Authors:** Wolfgang W Quitschke, Nicole Steinhauff, Jean Rooney

**Affiliations:** 1 Department of Psychiatry and Behavioral Science, Stony Brook University Medical Center, 100 Nicolls Road, Stony Brook, New York 11794-8101, USA; 2 Division of Laboratory Animal Resources, Stony Brook University Medical Center, 100 Nicolls Road, Stony Brook, New York 11794-8611, USA

## Abstract

**Introduction:**

Curcuminoids may improve pathological conditions associated with Alzheimer's disease. However, their therapeutic potential is limited by their exceedingly low bioavailability after oral administration. A method to deliver solubilized curcuminoids by injection was evaluated in Alzheimer transgenic mice.

**Methods:**

Amyloid protein precursor (APP)_SWE_, PS1dE9 mice were intravenously or subcutaneously injected at weekly intervals between the ages of 4 and 12 months with serum- or cyclodextrin-solubilized curcuminoids to assess their potential for plaque prevention. Alternatively, mice between the ages of 11 and 12 months were intravenously injected with cyclodextrin-solubilized curcuminoids at biweekly intervals to evaluate their ability to eliminate existing plaques. Plasma and brain levels of curcuminoids and their metabolites were also determined after subcutaneous and intravenous injection.

**Results:**

Weekly long-term injections did not result in a significant plaque load reduction. However, intravenous injection of cyclodextrin-solubilized curcuminoids at higher curcuminoid concentrations and at a biweekly frequency between the ages of 11 and 12 months reduced the plaque load to approximately 70% of the control value. After intravenous injection, plasma levels of 100 μM curcuminoids and brain levels of 47 nmol/g could initially be achieved that declined to essentially undetectable levels within 20 minutes. The primary curcuminoid metabolites in plasma were the conjugates of glucuronide or sulfate and hexahydrocurcuminoids as reduction products. In the brain, both hexahydrocurcuminoids and octahydrocurcuminoids were detected as major metabolites. After subcutaneous injection, maximal curcuminoid plasma levels of 23 μM and brain levels of 8 nmol/g were observed at 30 minutes after injection and curcuminoids remained detectable for 2 to 3 h.

**Conclusion:**

Curcuminoids are rapidly metabolized after injection and their effect on reducing plaque load associated with Alzheimer's disease may be dependent on the frequency of administration.

## Introduction

Curcumin is a yellow pigment extracted from the spice and coloring agent turmeric, where it occurs in amounts of 2 to 8% [1]. Commercial curcumin preparations typically contain a mixture of polyphenols, collectively referred to as curcuminoids. In addition to the primary component curcumin (CUR, 65 to 80%), they also contain smaller amounts of the co-extracted congeners demethoxycurcumin (DMC, 15 to 25%) and bisdemethoxycurcumin (BDMC, 5 to 15%) [2-4].

Curcumin binds to Aβ fibrils, presumably in the enol form [5], and stains amyloid plaques and neurofibrillary tangles in brain sections [6,7] and *in vivo *[8-10]. Curcumin inhibits Aβ fibril formation and promotes disaggregation of existing fibrils *in vitro *with IC_50 _values of 0.19 to 1 μM [9,11,12], although much higher IC_50 _values in the 10 to 12 μM range have been reported [13,14]. Curcumin similarly inhibits plaque formation or disrupts existing plaques in Alzheimer transgenic mouse models either after oral uptake [9,15-17] or intravenous (i.v.) injection [8]. Apart from the direct interaction with Aβ fibrils, curcuminoids may reduce plaque formation or ameliorate their effects by increasing Aβ uptake by macrophages [18], affecting amyloid protein precursor (APP) maturation [19], modulating APP processing enzymes [20,21], protecting neurons from Aβ induced toxicity [9,22-24] or influencing the expression of genes associated with apoptosis and inflammation [25]. Alternatively, curcumin degradation products may mediate similar effects (Review: [26]).

Despite such promising observations, the clinical use of orally administered curcuminoids is severely limited by their exceedingly low bioavailability, which is a direct consequence of their poor solubility in aqueous solutions and their rapid metabolic conversion (Reviews: [26-28]). To improve systemic availability, formulations containing high concentrations of curcuminoids were solubilized in either serum [29] or 2-hydroxypropyl-γ-cyclodextrin (HP-γ-CD) and injected into Alzheimer transgenic mice. The effect on plaque development, systemic availability and metabolism was investigated.

## Materials and methods

### Curcuminoid solubilization

Technical grade curcumin (Cayman Chemical Company, Ann Arbor, MI, USA) containing CUR (69%), DMC (19%) and BDMC (12%) was solubilized in either C57BL/6 mouse serum (Valley Biomedical, Winchester, VA, USA) or in an aqueous solution of 10% HP-γ-CD (Sigma-Aldrich, St. Louis, MO, USA) containing 0.6% NaCl, pH 6.8 by the sequential mixing with solid- and DMSO-dissolved curcumin as described elsewhere [29]. Briefly, 50 ml of serum or 10% HP-γ-CD was mixed by stirring with solid curcuminoids (50 mg/ml) for 16 h at 4°C. Thereafter, the suspension was clarified by centrifugation at 18,000 × g for 30 minutes. The supernatant was removed and DMSO-dissolved curcuminoids at a 1 M concentration were added (10 μl/ml) and again stirred for 16 h. The resulting suspension was then clarified by two successive centrifugations at 18,000 × g. The supernatant was sterilized by filtration through a 0.45 μm membrane filter (Pall Corporation, Ann Arbor, MI, USA). The final supernatants contained either 3-4 mM total curcuminoids solubilized in serum or 24 mM curcuminoids solubilized in 10% HP-γ-CD (full-strength).

### Animals, injections and tissue preparation

For the study on plaque prevention, female APP_SWE_, PS1dE9 transgenic mice (Jackson Laboratories, Bar Harbor, ME, USA) were i.v. injected with serum-solubilized curcumin via the tail vein once per week starting at four months of age. The average weight of the mice was about 25 g (range: 23 to 27 g) and they were injected with a total volume of 0.1 ml serum-solubilized curcuminoids. This protocol was discontinued after five injections (four weeks) due to adverse reactions. As an alternative, full-strength HP-γ-CD-solubilized curcuminoids were diluted four-fold with isotonic saline to a concentration of 6 mM and similarly injected until the mice had reached the age of about 10.5 months. For the remaining six weeks, the mice were subcutaneously (s.c.) injected between the shoulder blades at weekly intervals with 0.4 ml full-strength HP-γ-CD-solubilized curcuminoids until the final age of 12 months. For the study on plaque resolution, mice at 11 months of age were i.v. injected for 1 month twice weekly (nine injections) with 0.1 ml of full-strength HP-γ-CD-solubilized curcuminoids. The brains from a total of 23 mice were successfully processed for histological evaluation. These included one negative control without transgenes, three positive controls without injections, three positive controls each with injections for plaque prevention and plaque resolution, six experimental mice injected for plaque prevention and seven mice for plaque resolution.

Brain and plasma levels following curcuminoid injection were analyzed in wild type C57BL/CH3, transgenic C57BL/CH3 and CV-1 mice. Since the CV-1 mice were larger with an average weight of about 50 g (range: 45 to 55 g), these were i.v injected with 0.2 ml or s.c. injected with 0.8 ml of full-strength HP-γ-CD-solubilized curcuminoids to account for the larger mass and blood volume. After injection, mice were sacrificed by exposure to CO_2 _at selected time intervals. Blood (approximately 1 ml) was collected by cardiac puncture into tubes containing EDTA and the tissues were subsequently removed and stored at -75°C until further processing. The blood was centrifuged at 14,000 × g for one minute for plasma collection.

To estimate the maximum uptake/binding of curcuminoids in the brain under the applied i.v. injection conditions, one CV-1 mouse was exposed to curcuminoids by cardiac perfusion, as modified from the procedure described below. The mouse was sequentially perfused with 10 ml mouse serum, 30 ml mouse serum containing 5% full-strength HP-γ-CD-solubilized curcuminoids, and then washed with 30 ml of PBS.

All procedures involving animal handling and processing were approved by the Stony Brook University Institutional Animal Care and Use Committee (IACUC) in compliance with the National Institutes of Health guidelines.

### Amyloid plaque staining of brain sections and quantitative analysis

At the age of 12 months, all mice were prepared for histological evaluation. Terminal deep anesthesia was induced by s.c. injection of 0.2 ml of pentobarbital (50 mg/ml). The chest cavity was exposed and a blunt syringe needle inserted into the left ventricle of the heart. The right atrium was cut open and the mice were sequentially perfused with 10 ml of wash solution (NaCl, 8 g/L; dextrose, 4 g/L; anhydrous CaCl_2_, 0.23 g/L; sodium cacodylate trihydrate, 0.34 g/L) and 30 ml of fix solution (sucrose, 40 g/L; paraformaldehyde, 40 g/L; sodium cacodylate, 14.34 g/L; pH 7.2). The whole heads stripped of skin and eyes were placed in 30 ml of sodium cacodylate buffer (0.2 M, pH 7.4) and shipped to NeuroScience Associates (Knoxville, TN, USA) for further processing.

Brains were removed from skulls and treated with 20% glycerol and 2% dimethylsulfoxide to prevent freeze-artifacts, and embedded in a gelatin matrix using MultiBrain Technology^® ^(NeuroScience Associates). After curing, the block of embedded brains was rapidly frozen by immersion in isopentane chilled to -70°C with crushed dry ice, and mounted on a freezing stage of an AO 860 sliding microtome. The MultiBrain^® ^block was sectioned coronally at 35 μm. Sections were sequentially collected into 24 containers that were filled with Antigen Preserve solution (49% PBS pH 7.0, 50% ethylene glycol, 1% polyvinyl pyrrolidone). At the completion of sectioning, each container held a serial set of 1-of-every-24th section (one section every 840 μm). Each of the MultiBrain^® ^sections cut from the block was a composite holding individual sections from each of the brains embedded in the block. With such composite sections, uniformity of staining was achieved across treatment groups.

Brain sections were silver-stained according to the Campbell-Switzer Alzheimer's method for plaques and tangles [30,31]. Overall, this stain displays sensitivity and specificity properties comparable to immunohistochemical methods [32-35]. MultiBrain^® ^sections were collected and stained free-floating at room temperature. The sections were placed in freshly prepared 2% ammonium hydroxide for five minutes, and then transferred sequentially to a silver-pyridine-carbonate solution for 40 minutes, 1% citric acid for 3 minutes and 0.5% acetic acid until ready for development. The sections were developed in Physical Developer ABC solution (after Gallyas; containing sodium carbonate, citric acid, tungstosilicic acid and formaldehyde) with the development time being visually assessed. The development was stopped by placing the sections briefly in 0.5% acetic acid. Sections were mounted on gelatinized (subbed) glass slides, dehydrated and cover-slipped.

The Alzheimer's disease (AD) plaque burden was determined by quantifying and averaging the plaque area percentage of cortical area from four sections per animal. The coronal section containing the full anterior commissure was chosen as the first section to quantify, and the subsequent three stained sections completed the set of four. Individual slides containing an entire MultiBrain^® ^section were scanned at 4X as high-resolution 24 bit color tif files. The color density of the images was adjusted in Photoshop^® ^to provide contrast between the plaques and the background, but without using Brightness-Contrast as this function changes the size of the plaques. The threshold of each image was determined to provide an image with plaques and no background in ImageJ (NIH, Bethesda, MD, USA). Using the threshold adjustment, a level was determined that removed the background while leaving plaques intact. Plaque size and number was quantified in ImageJ with Size = 0 to infinity and circularity = 0 to 1. From Analyze > Particles, the result was copied into a spreadsheet for the appropriate level of the image (1 to 4). This process was repeated for all images. Percent plaque area was calculated in Excel^® ^and graphed in SigmaPlot™ (11.2) according to animal number and plaque density.

### Tissue and plasma extraction for reversed phase chromatography

Approximately 200 mg of brain tissue (approximately one hemisphere) was homogenized in 0.7 ml of 50% acetonitrile, 0.01% ammonium acetate, pH 4.5 (total volume approximately 0.9 ml). The homogenate was transferred to a 1.5 ml microcentrifuge tube and centrifuged for five minutes at 18,000 × g. Subsequently, 0.4 ml of the supernatant was removed and transferred to a fresh microcentrifuge tube, which was then mixed with 0.8 ml of acetone and left for 12 to 16 h at -20°C. The precipitated protein was centrifuged at 14,000 × g for five minutes and the clear supernatant was transferred to a fresh tube and evaporated under vacuum. The dry residue was re-solubilized in 0.2 ml 37.5% acetonitrile, 0.01% ammonium acetate, pH 4.5 and clarified by centrifugation and the supernatant was loaded onto a reversed phase column and separated as described elsewhere [29,36]. Plasma (0.2 ml) was mixed with an equal volume of 50% acetonitrile, acetone precipitated and then processed the same way as the brain tissue.

### Analysis of curcuminoids and metabolites

Curcuminoids and their conjugated metabolites were separated by reversed phase chromatography and monitored at an absorption wavelength of 427 nm. Curcuminoids were quantitated based on standard curves for curcumin (analytical grade) by integration of the peaks with the FPLC Unicorn™ (version 5.10) program (GE Healthcare Life Sciences, Piscataway, NJ, USA). The molar absorptivities (ε) of the three curcuminoids dissolved in ethanol were shown to range from 6.73 (x 10^4 ^L cm^-1^mol^-1^) for CUR, 5.78 for DMC and 4.95 for BDMC at 425 nm [37]. Since the molar absorptivity for curcumin dissolved in acetonitrile was essentially the same as in ethanol, the integrated values of the DMC and BDMC peaks were multiplied by the respective factors 1.17 and 1.36 to obtain molar concentrations in all experiments. The conjugated metabolites were identified by mass spectrometry as described elsewhere [36] and they were assumed to have the same molar absorption as their parental counterparts [38]. The reductive metabolites of curcuminoids were visualized at an absorption wavelength of 280 nm or 310 nm. The molar absorptivities of the hexa- and octahydrocurcuminoids were taken from the data presented by Hoehle *et al. *[39], which were in excellent agreement with standard curves generated with commercial preparations of hexahydrocurcumin (Sigma-Aldrich) and octahydrocurcumin (Sabinsa Corporation, East Windsor, NJ, USA). Unless otherwise indicated, all data points were calculated as the average of 3 to 10 independent experiments. Error bars represent the standard deviation from the mean.

### Curcuminoid binding to NT2/D1 cells

NT2/D1 cells were grown in 25 cm^2 ^flasks to a density of about 90% confluence (6 to 8 × 10^6 ^cells) with 6 ml of Dulbecco's Minimal Essential Medium (DMEM) containing 5% fetal calf serum (FCS). For curcuminoid dose curves to determine binding dissociation constants, media were prepared with increasing amounts (10 μl to 2 ml) of curcuminoids solubilized in mouse serum or 10% HP-γ-CD and supplemented with DMEM for a total volume of 6 ml. Media for serum competition curves were prepared by adding 300 μl of solubilized curcuminoids to a total volume of 6 ml. The remaining volume consisted of varying ratios of DMEM and mouse serum to generate dose curves with constant curcuminoid and variable serum concentrations. Curcuminoids solubilized in mouse serum at a 4 mM initial concentration yielded a final media concentration of 200 μM. Curcuminoids solubilized in HP-γ-CD were either used full-strength (24 mM) or diluted with 0.6% saline to a 6 mM concentration, resulting in final media concentrations of 1.2 mM and 300 μM, respectively. After 1 h incubation, 0.4 ml of medium was withdrawn for the determination of free curcuminoids and mixed with 0.8 ml of acetone. Cells were then washed three times in the flasks with 5 mL of 0.9% saline and then scraped into 1 ml of the same solution. The cell suspension was centrifuged at 3,000 × g for one minute and the supernatant removed. The cell pellet was resuspended in 1 ml of 0.6% saline and re-centrifuged. The final pellet was suspended in 0.4 ml of 0.6% saline and mixed with 0.8 ml of acetone. All samples were stored at -20°C for 2 to 16 h and processed for reversed phase chromatography as described above for tissue preparation. After curcuminoid quantitation, data points were combined from three independent experiments, normalized to 10^6 ^cells, and fitted to a ligand binding function (y = (B_MAX_*x)/(K_D_+x)+N_S_x) or a hyperbolic decay function (y = ab/(b+x)) using SigmaPlot™(11.2) as described elsewhere [36].

## Results

### Curcuminoids solubilized in serum and HP-γ-CD

**Figure 1:**
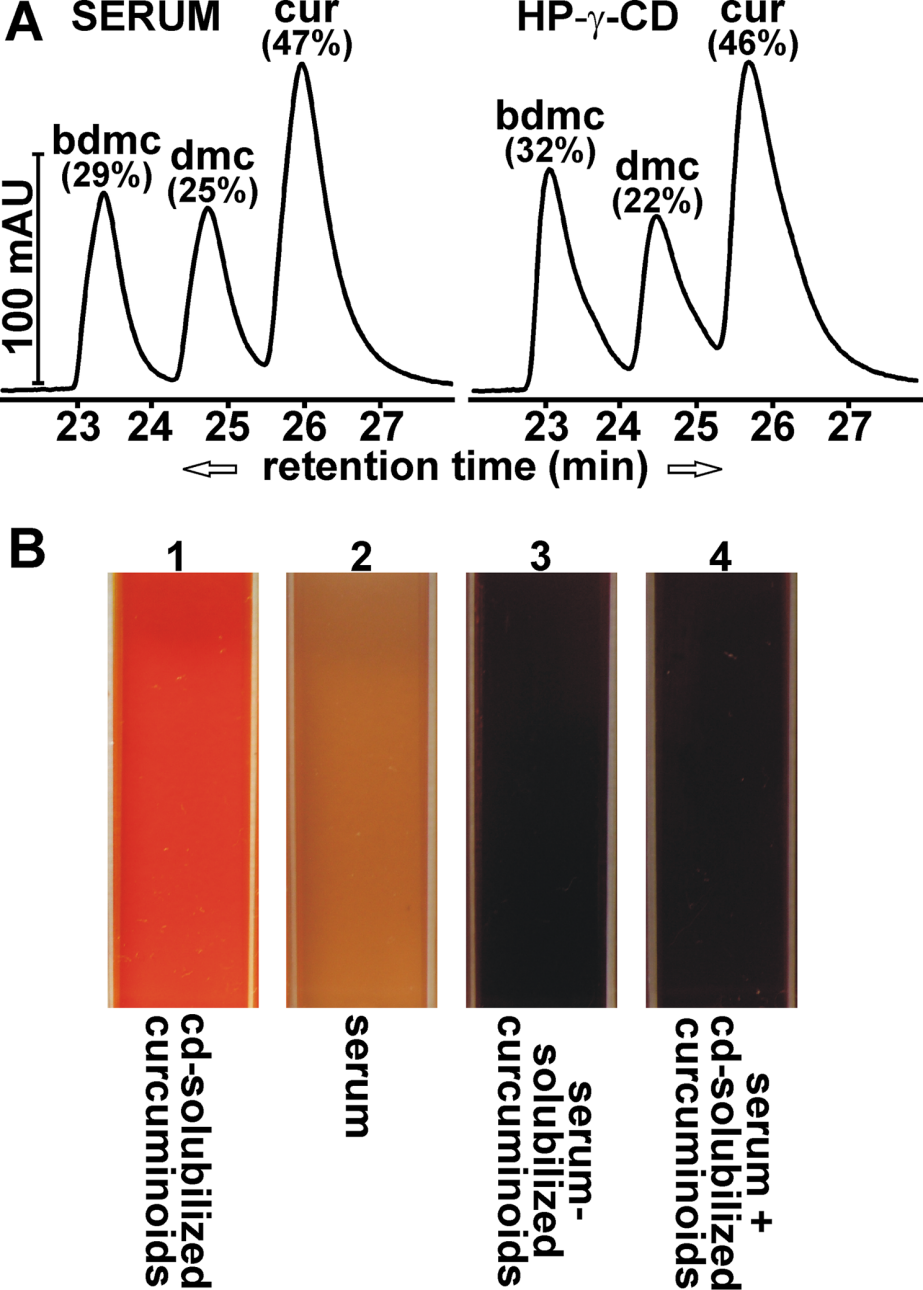
**Curcuminoid solubilization**. **(A) **Chromatograms of curcuminoids solubilized either in serum (left panel) or 10% HP-γ-CD (right panel). The relative contribution of each curcuminoid (%) relative to the total amount (100%) is indicated. **(B) **The color of curcuminoids solublized in 10% HP-γ-CD (1) compared to normal serum (2), curcuminoids solubilized directly in serum (3), and HP-γ-CD-solubililized curcuminoids diluted to 5% with serum (4).

To assess the use of solubilized curcuminoids as a therapeutic or preventative agent in the treatment of Alzheimer's disease, technical grade curcumin was initially solubilized in mouse serum. Technical grade curcumin is the type of preparation used in the vast majority of studies on this compound and it contains all three curcuminoids as they are represented in the turmeric powder. All three curcuminoids were included in this study to neutralize any effects due to their differential chemical stability, metabolic conversion or cellular binding affinity [29,36]. Furthermore, all three curcuminoids have been shown to inhibit Aβ fibrillogenesis and they may complement each other to inhibit the process that leads to plaque formation [40]. The sequential solubilization of solid and DMSO-dissolved curcumin yielded a total soluble concentration of 3-4 mM and a curcuminoid distribution as depicted in Figure 1A. The feasibility of using such serum-solubilized curcuminoids for i.v. injection was initially tested on Sprague-Dawley rats using serum derived from random animals of the same strain. Rats were injected weekly via the tail vein with serum-solubilized curcuminoids at approximately 7% of the total blood volume for up to four months without adverse effects.

A similar injection protocol was adapted for the Alzheimer transgenic mouse strain APP_SWE_, PS1dE9, which is a hybrid from a cross between C_57_BL/6 and C_3_H mice [41]. Since serum from such a cross is commercially unavailable, serum derived from C_57_BL/6 mice was used. The first two injections proceeded uneventfully. However, after the third injection adverse reactions occurred that were consistent with anaphylaxis. Since such reactions were also observed in control mice injected with serum devoid of curcuminoids, it was concluded that these were caused by incompatible serum components. Most mice recovered within one hour; however, some fatalities occurred. It was, therefore, deemed impossible to continue with such serum injections and a new vehicle for the solubilization of injectable curcumin had to be found.

Solubilizing curcuminoids in 10% HP-γ-CD proved to be ideal for this purpose. Mixing either solid curcuminoids or DMSO-dissolved curcuminoids with 10% HP-γ-CD, preferentially solubilized BDMC or CUR, respectively. As such, the distribution of individual curcuminoids followed the same pattern as that observed with serum or albumin solutions [29]. To maximize the amount of curcuminoids solubilized and to limit the final concentration of DMSO to 1%, curcuminoids were added sequentially, first as a solid powder followed by DMSO-dissolved curcuminoids. This resulted in a curcuminoid distribution that was very similar to that obtained with serum by the same procedure (Figure 1A), albeit with a higher final curcuminoid concentration of approximately 24 mM. Upon diluting such curcuminoid solutions with serum, no precipitation occurred that might have had adverse effects on the circulation. In addition, the HP-γ-CD solubilized curcuminoids partially equilibrated with serum components as evidenced by a shift in color from yellow to deep red, which is similar to the color of curcuminoids directly solubilized in serum (Figure 1B). For long-term i.v. injection studies, the full-strength HP-γ-CD-solubilized curcuminoids were diluted four-fold to a final concentration of 6 mM curcuminoids in 2.5% HP-γ-CD. This resulted in a curcuminoid concentration similar to that obtained by serum-solubilization, which caused no adverse effects. In addition, the amount of HP-γ-CD (100 mg/kg) injected was below that considered toxic for chronic exposure [42].

### The effect of injecting soluble curcuminoids on plaques in Alzheimer transgenic mice

**Figure 2:**
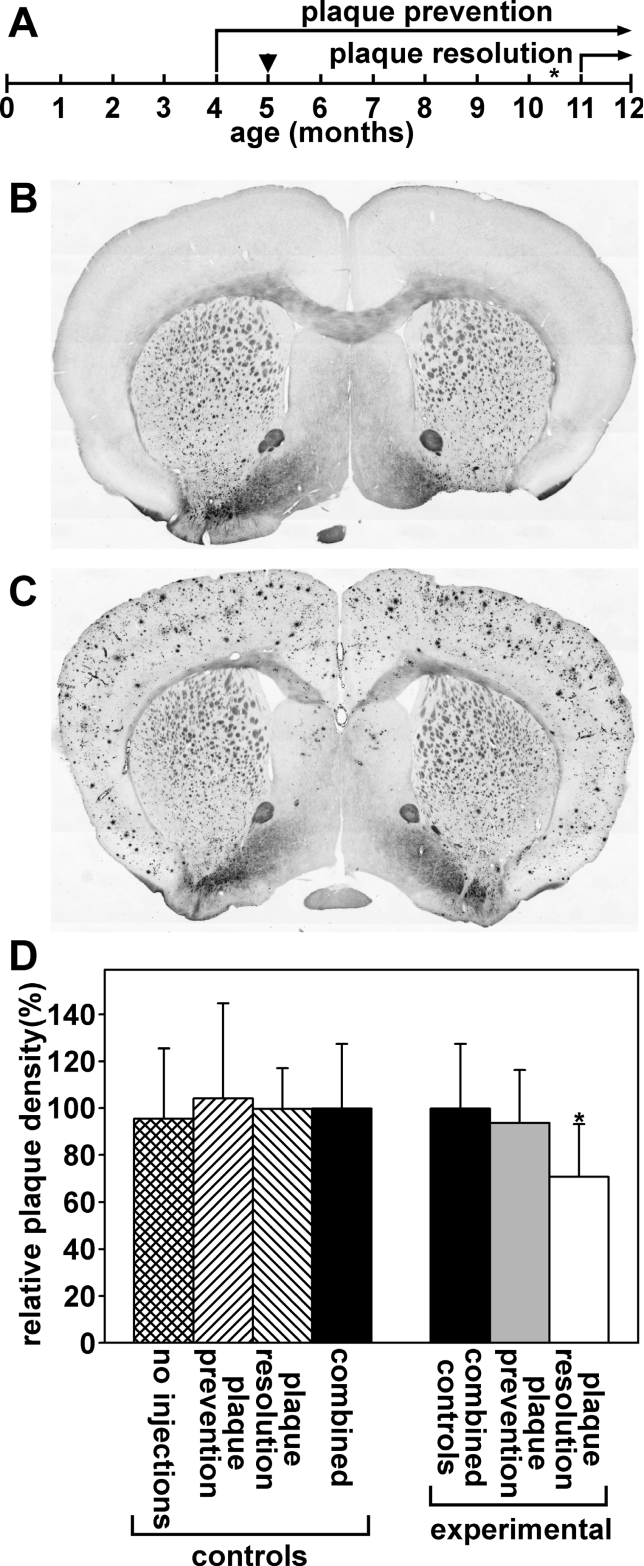
**Amyloid plaques in brains of mice and the effect of curcuminoid injections**. **(A) **Time course of curcuminoid injections into Alzheimer transgenic mice. Arrowhead denotes the time point when serum-solubilized curcuminoids were switched to HP-γ-CD-solubilized curcuminoids, asterisk when i.v. injections were replaced with s.c. injections. **(B) **Silver stained brain section from a control non-transgenic mouse without plaques. **(C) **Comparable section from an Alzheimer transgenic mouse at the age of 12 months. **(D) **The effect of curcuminoid injections on amyloid plaque load. Left panel: Alzheimer transgenic control mice were analyzed at 12 months of age and received either no injections (*n *= 3), or injection of vehicle alone according to the protocols for plaque prevention (*n *= 3) or plaque resolution (*n *= 3). The differences in the relative plaque levels in each of the groups were not statistically different and the values were combined and assigned the value of 100% (*n *= 9). Right panel: The combined control group was compared to experimental mice that had been injected for plaque prevention (*n *= 6) or plaque resolution (*n *= 7) with a statistical difference defined at the level of *P *< 0.05 (*P = 0.0496).

The transgenic mouse line APP_SWE_, PS1dE9 contains the Swedish mutations (K595N/M596L) within a chimeric mouse/human APP695, and the human transgene for presenilin 1 with a deletion of exon 9 [41]. This mouse line begins to develop plaques at 4 to 6 months of age, which progresses to extensive plaque load by 12 months of age [43]. The ability of curcuminoids to prevent amyloid plaque formation was studied by weekly tail vein injections. Injections of 0.1 ml serum-solubilized curcuminoids (4 mM) were started at the age of four months and replaced with HP-γ-CD-solubilized curcuminoids (6 mM) four weeks later. At the age of about 10.5 months, the tail veins of many mice had deteriorated to the point that further i.v. injections were rendered impossible. For the last six weeks, until the age of 12 months, tail vein injections were, therefore, replaced with weekly s.c. injections of 0.4 ml full-strength (24 mM) HP-γ-CD-solubilized curcuminoids. The ability of curcuminoids to resolve existing plaques was investigated by tail vein injection twice weekly with 0.1 ml of 24 mM HP-γ-CD-solubilized curcuminoids. Nine injections were carried out between the ages of 11 and 12 months (Figure 2A).

The i.v. injection of 6 mM HP-γ-CD-solubilized curcuminoids caused no obvious detrimental effects. However, within seconds after i.v. injection of curcuminoids at 24 mM concentrations, acute reactions were observed that included trembling and agitation followed by a period of lethargy. These reactions subsided within five minutes after injection and caused no deaths in otherwise healthy animals. Such effects were not observed in control animals and it was concluded that curcuminoids were toxic at these high concentrations. The rapid resolution of symptoms is consistent with the notion that curcuminoids are rapidly metabolized. No adverse effects were noted after s.c. injections.

At the age of 12 months, transgenic mice showed vigorous amyloid plaque development compared to a negative control not carrying the transgene (Figure 2B, C). The plaque load in the experimental groups was determined for plaque prevention (*N *= 6) and for plaque resolution (*N *= 7). Included were also positive control mice that had not received any injections (*N *= 3), and mice from each group (*N *= 3) that had received injections of the solubilization vehicle without curcuminoids. The average plaque loads between the three control groups showed no significant differences. For statistical purposes, the three control groups were, therefore, combined (*N *= 9) and compared with the experimental groups (Figure 2D). The plaque load of mice that had received weekly injections of curcuminoids for eight months for plaque prevention, showed no significant difference from the control group, although the overall average was slightly reduced. Mice that received bi-weekly injections of curcuminoids at a four-fold higher concentration for four weeks for plaque resolution, had a statistically significant (*P *< 0.05) lower plaque load at approximately 70% of the control value (Figure 2D).

These results demonstrate that sporadic injections of curcuminoids did not result in the panacea initially hoped for. To design more effective injection protocols with the goal of achieving a more robust response, the fate of curcuminoids after both i.v. and s.c. injections was further examined.

### Curcuminoid levels in plasma and brain after intravenous and subcutaneous injection

**Figure 3:**
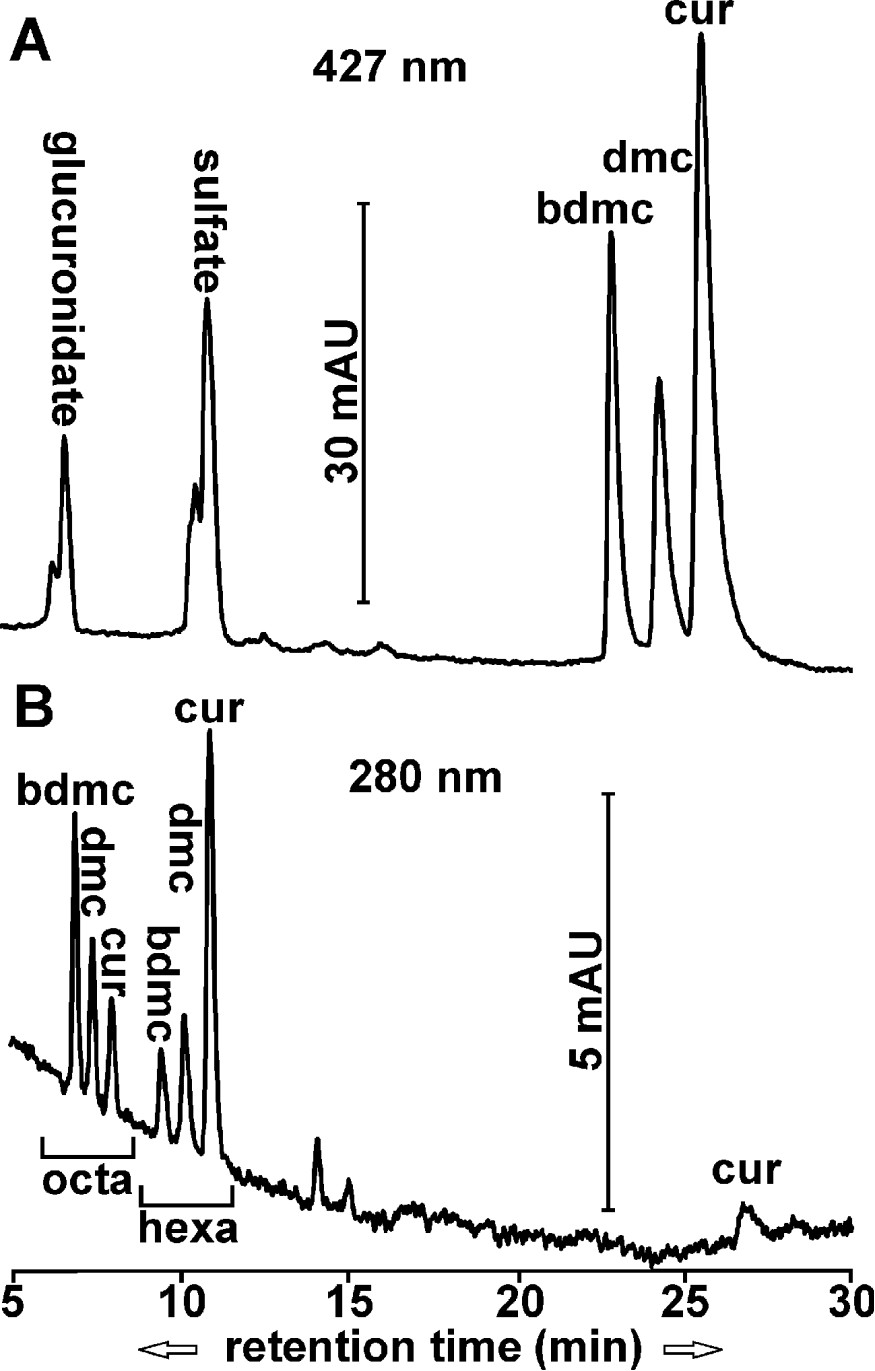
**Elution profiles of curcuminoids and metabolites in mice after i.v. injections**. **(A) **Chromatogram (427 nm) of curcuminoids and monoconjugates of sulfate and glucuronide extracted from plasma five minutes after injection. **(B) **Chromatogram (280 nm) of octahydro- and hexahydrocurcuminoids extracted from brain 15 minutes after injection.

After i.v. injection, curcuminoids and selected metabolites were measured in plasma and brain (Figure 3) from both wild type and transgenic C57BL/CH3, as well as CV-1 mice. Since the data from both strains of mice were indistinguishable beyond what would be attributable to individual variations, the results were combined for statistical purposes. Unmodified curcuminoids and their sulfate and glucuronide conjugates were detected in plasma at a 427 nm absorption wavelength (Figure 3A). The hexa- and octahydrocurcuminoid reduction products were detected in both plasma and brain at the 280 nm wavelength (Figure 3B). Significant amounts of di- and tetrahydrocurcuminoids were not observed. In addition, conjugates of reduction products or diconjugate curcuminoids could not be reliably determined, either because their concentration was too low or their absorption peaks overlapped with those from numerous co-extracted unrelated compounds that eluted close to the front.

**Figure 4:**
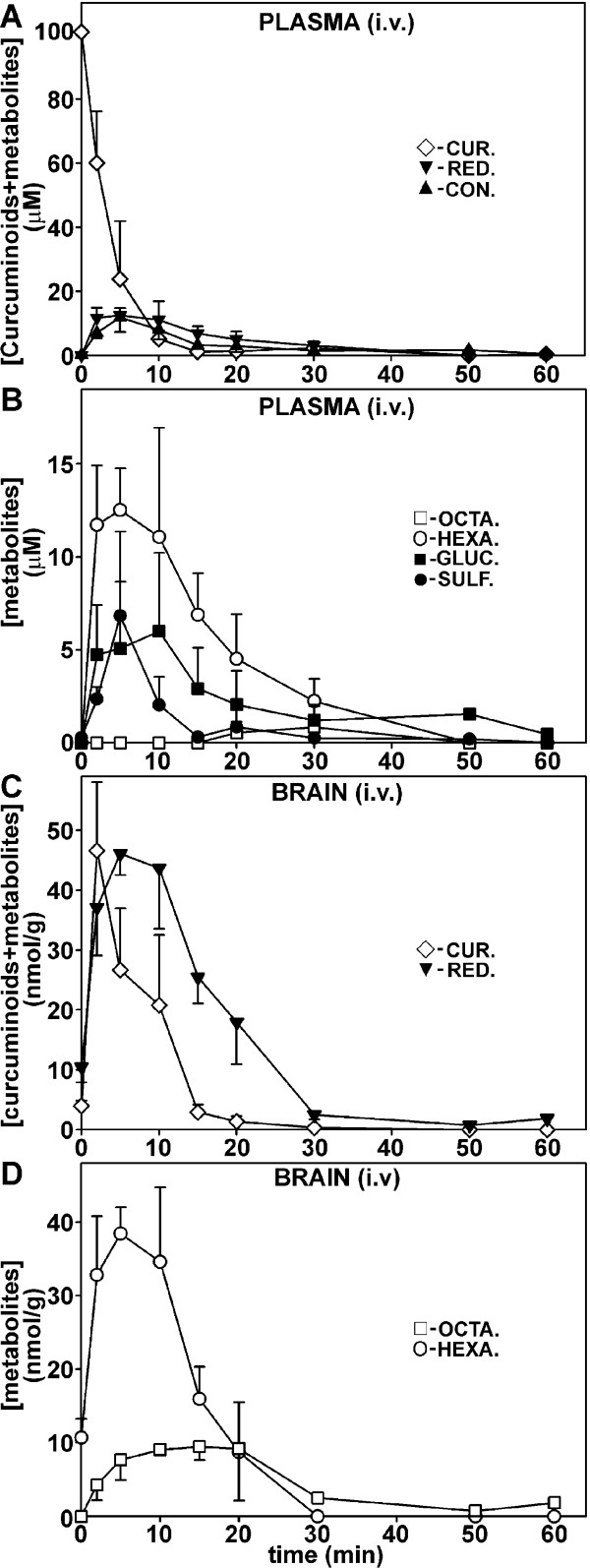
**Concentration of curcuminoids and metabolites in plasma (uM) and brain (nmol/g) after i.v. injection**. **(A) **Time course of total curcuminoids (◊), reduction products (hexa- + octacurcuminoids, ▼), and monoconjugates (sulfates + glucuronides, ▲) in plasma. **(B) **Time course of individual metabolite concentrations in plasma: hexahydrocurcuminoids (○), octahydrocurcuminoids (□), curcuminoid monoglucuronides (■), and curcuminoid monosulfates (●). **(C) **Time course of total curcuminoids (◊) and reduction products (▼) in brain. **(D) **Time course of hexahydro- (○) and octahydrocurcuminoids (□) in brain.

Initial plasma concentration of curcuminoids reached a high level of approximately 100 μM immediately following i.v. injection (Figure 4A), which declined rapidly to essentially undetectable levels within 20 minutes. Both conjugation and reduction products were also transiently observed in plasma at concentrations below 20 μM (Figure 4A, B). Curcuminoid sulfates and glucuronides in plasma occurred concurrently in individual mice, but their relative levels varied widely between animals and time after injection. Overall, the level of sulfates peaked early and declined to low levels by 15 minutes while glucuronides persisted longer and were detected for up to 1 h. The primary reduction products in plasma were hexahydrocurcuminoids and these persisted for at least 30 minutes. Octahydrocurcuminoids were detected transiently at low levels between 20 and 30 minutes after injection (Figure 4B).

**Figure 5:**
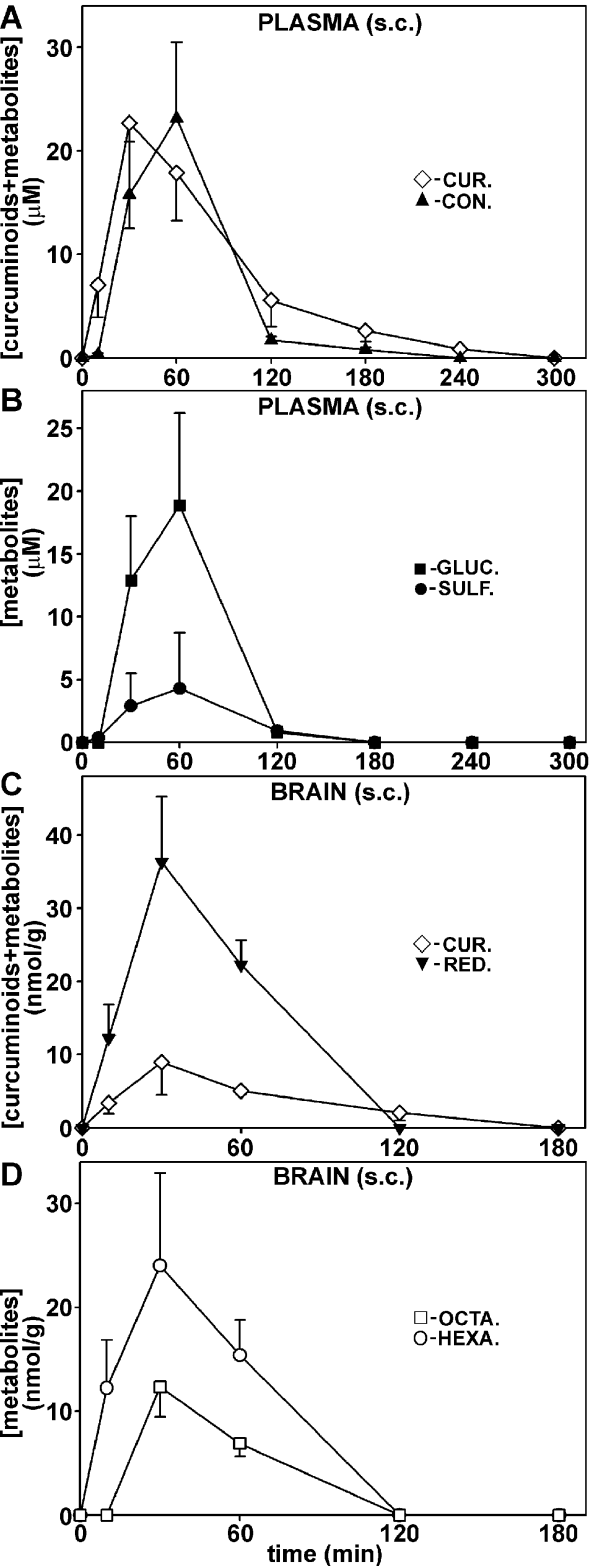
**Concentration of curcuminoids and metabolites in plasma (μM) and brain (nmol/g) after s.c. injection**. **(A-D) **Same as depicted in Figure 4, except that significant concentrations of curcuminoid reduction products were not detected in plasma.

After s.c. injection, peak plasma curcuminoid levels were delayed compared to i.v. injection and they gradually reached a maximum after 30 minutes at 23 μM. Lower levels of curcuminoids (approximately 1 μM) could be detected for up to 4 h. The peak level of curcuminoid conjugates occurred with a delay compared to the parental compounds at 1 h after s.c. injection (Figure 5A). The primary monoconjugates were consistently glucuronides, which at peak levels were about five-fold higher than sulfates (Figure 5B). Significant amounts of reduction products were not observed after s.c. injection.

In brain tissue, the highest levels of curcuminoids (approximately 47 nmol/g) occurred with a slight delay compared to plasma at two minutes after i.v. injection. No curcuminoid conjugates were observed, which indicated that contamination with blood was insignificant. While the concentration of curcuminoids declined to undetectable levels after 30 minutes, the reduction products could be detected for up to 1 h (Figure 4C). Hexahydrocurcuminoids were the main reduction products in the brain, with peak concentrations reaching approximately 40 nmol/g at five minutes after injection. The levels of octahydrocurcuminoids were lower but persisted for up to 1 h (Figure 4D).

After s.c. injection, brain curcuminoid levels were considerably lower (approximately 8 nmol/g) than after i.v. injection (Figure 5C) with a maximum attained at 30 minutes. However, levels above 1 nmol/g were observed for more than 2 h, whereas similar concentrations were already reached within 20 minutes after i.v. injection. In contrast, the levels of reduction products (approximately 36 nmol/g) were comparable to those observed after i.v. injection (approximately 46 nmol/g). Similar to i.v. injection, octahydrocurcuminoids appeared with a slight delay compared to hexahydrocurcuminoids. Nevertheless, all reduction products were essentially undetectable after 2 h following s.c. injection (Figure 5D).

This demonstrates that significant levels of curcuminoids can initially be achieved in plasma and brain after both i.v. and s.c. injection. However, these levels are transient and although the curcuminoid levels decline somewhat more slowly after s.c injection, ultimately they persist at detectable concentrations for no longer than two to four hours. These results further suggest extensive binding of injected curcuminoids to cells and tissues, followed by rapid local metabolic conversion and gradual release into the circulation. In addition, the time course of the appearance of metabolites in the brain indicates sequential reduction from hexahydrocurcuminoids to octahydrocurcuminoids and this aspect was examined in further detail.

### Sequential generation of curcuminoid reduction products in brain after i.v. injection

**Figure 6:**
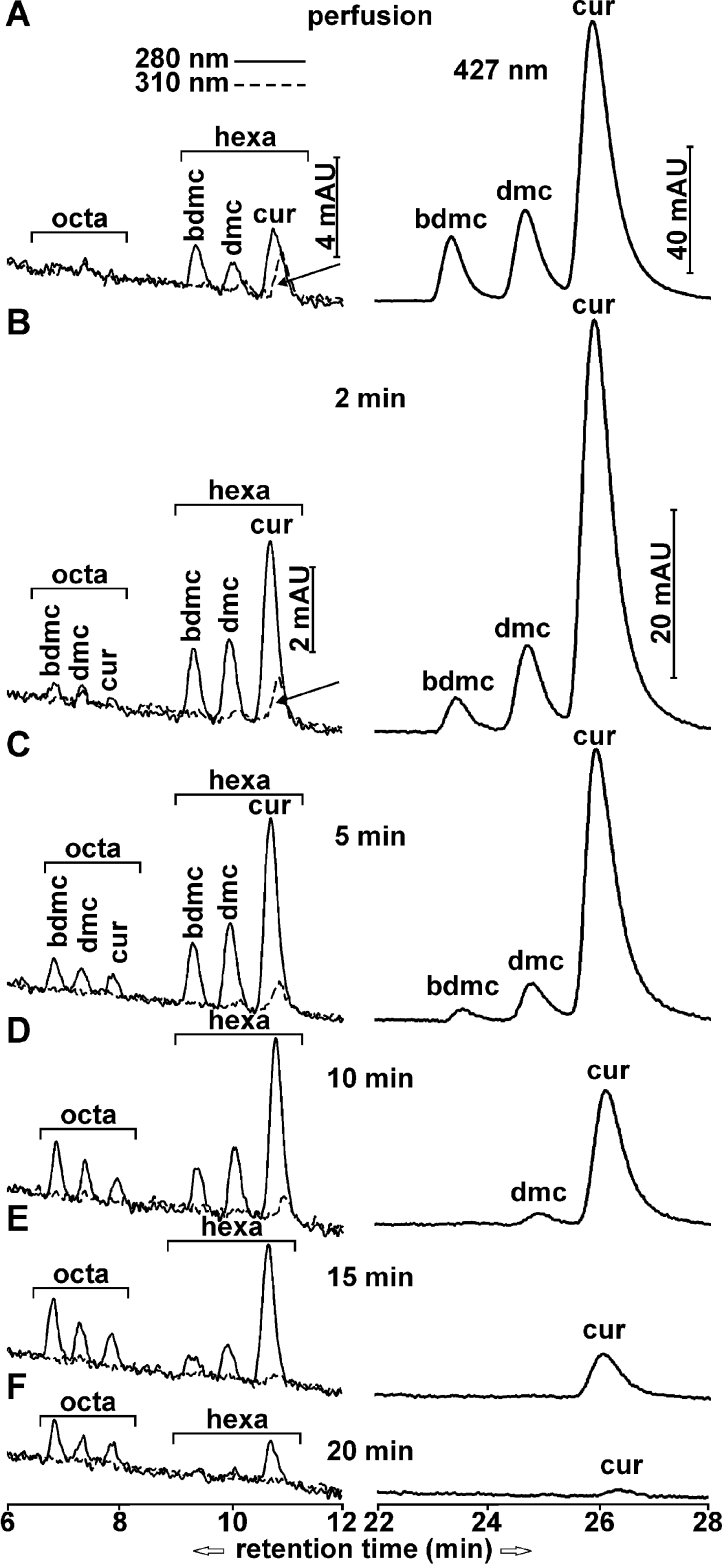
**Chromatograms of curcuminoids and reduction products in brain after cardiac perfusion and i.v. injection**. Curcuminoids (right panels, retention time 22 to 28 minutes) were monitored at wavelength 427 nm and reduction products (left panels, retention time 6 to 12 minutes) at wavelengths 280 nm (solid lines) and 310 nm (dashed lines). The position of hexa- and octahydrocurcuminoids are indicated by brackets. **(A) **Elution profiles after cardiac perfusion. **(B-F) **Elution profiles at 2 minutes (B), 5 minutes (C), 10 minutes (D), 15 minutes (E) and 20 minutes (F) after i.v. injection.

**Figure 7:**
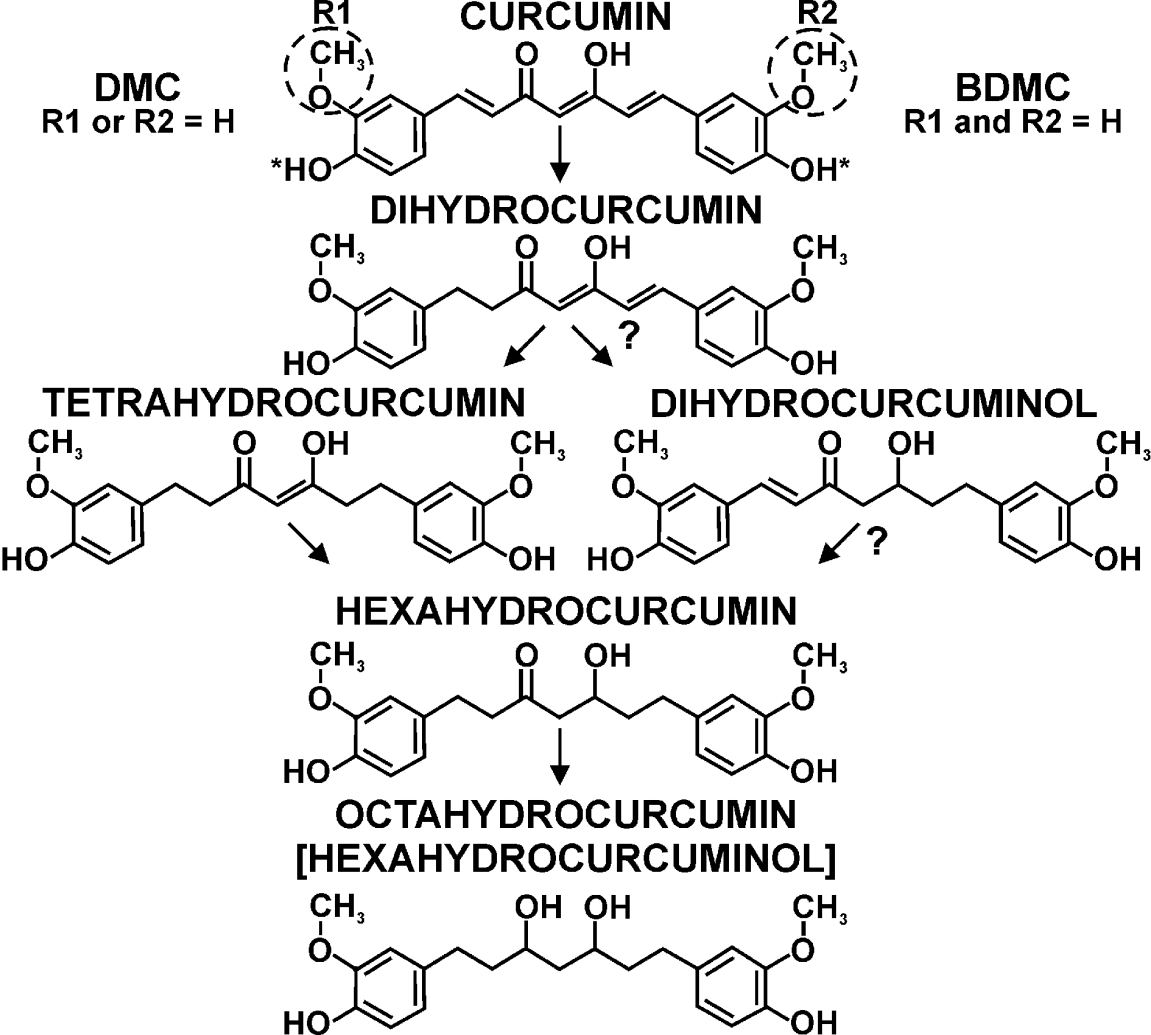
**Structure of curcuminoids and putative alternative reduction pathways**.

It takes at least several minutes before brains can be removed and cooled to the point that further metabolism may be considered negligible. To investigate how postmortem processes contribute to the metabolism of curcuminoids, a mouse was subjected to cardiac perfusion with HP-γ-CD-solubilized curcuminoids diluted with serum to a similar concentration as that achieved by i.v injections (5%). The total amount of curcuminoids extracted from a brain after perfusion was approximately 256 nmol/g and this may be considered the maximum capacity for uptake/binding within these concentration parameters. Comparable values in other organs were 1,383 nmol/g for the liver, 579 nmol/g for the kidney and 287 nmol/g for the small intestine. The primary reduction products observed in brains after perfusion were hexahydrocurcuminoids at a concentration of approximately 16 nmol/g and no quantifiable amounts of octahydrocurcuminoids (Figure 6A). A similar pattern was observed in the other organs, except that a small additional amount of glucuronidation was detected in the liver (not shown). This suggests that postmortem reduction is blocked at the hexahydrocurcuminoid stage. However, in addition to the hexahydrocurcuminoid peaks at 280 nm wavelength, further peaks were observed at 310 nm wavelength. The tentative identity of these compounds was previously established in an embryonal carcinoma cell line [36] and they are here designated as dihydrocurcuminoidols, which may represent alternative intermediates in the curcuminoid reduction pathway (Figure 7)

Under *in vivo *conditions, the brain content of curcuminoids declined to essentially undetectable levels within 20 minutes after i.v. injection, concomitant with a sequential accumulation of reduction products. Hexahydrocurcuminoids appeared rapidly within 2 minutes and reached a maximum after 5 to 10 minutes. In contrast, octahydrocurcuminoids emerged later with peak concentrations after 10 to 15 minutes. The relative levels of hexahydrocurcuminoids reflected those of the parental curcuminoids in the succession CUR > DMC > BDMC, whereas the relative levels of octahydrocurcuminoids were reversed. Dihydrocurcuminoidols, were also detected here, albeit at lower relative levels (Figure 6B-F).

### Differential cellular curcuminoid binding upon solubilization in serum or HP-γ-CD

**Figure 8:**
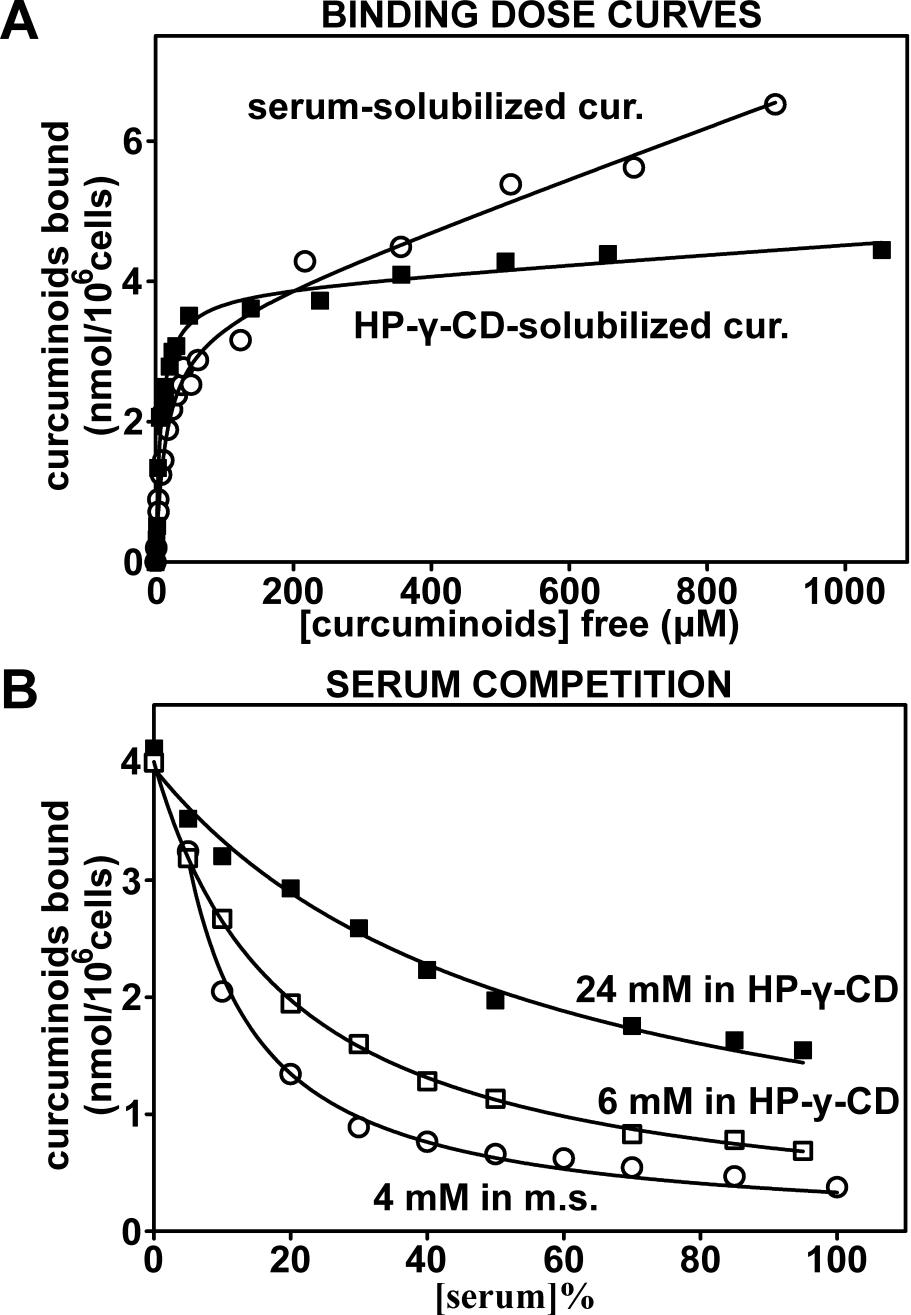
**The binding of curcuminoids solubilized in mouse serum or HP-γ-CD to NT2/D1 cells**. **(A) **Binding dose curves of curcuminoids solubilized in either mouse serum (O) or 10%-γ-CD (■). NT2/D1 cells were incubated with increasing concentrations of curcuminoids and the concentration of unbound (free) and the amount of cellular bound curcuminoids was determined. From the resulting dose curves the following binding parameters were determined: K_D_: 14.87 ± 1.97 μM (O), 7.58 ± 0.92 μM (■); B_MAX_: 3.37 ± 0.15 nmol/10^6 ^cells (O), 3.87 ± 0.15 nmol/10^6 ^cells (■). **(B) **Binding dose curves with constant concentrations of curcuminoids added at a ratio of 1:20 (5%) to media containing variable serum concentrations (0 to 100%). Curcuminoids were solubilized in HP-γ-CD at a concentration of 24 mM (final concentration in medium: 1.2 mM; ■), diluted four-fold to 6 mM with 0.6% NaCl (final concentration in medium: 300 μM; □), or solubilized in mouse serum at 4 mM (final concentration in media: 200 μM; O). The data points were fitted to a hyperbolic decay function (y = ab/(b + x)).

Cellular curcuminoid binding as it is affected by the solubilization vehicles and their dilution with mouse serum was evaluated in cultured NT2/D1 cells, in which these parameters have been extensively characterized [36]. Binding dose curves were generated by incubating NT2/D1 cells with increasing amounts of curcuminoids solubilized in either mouse serum or 10% HP-γ-CD. The binding dose curves yielded an apparent K_D _of 14.88 μM for mouse serum- and 7.58 μM for HP-γ-CD-solubilized curcuminoids (Figure 8A). A similar dose curve established with curcuminoids solubilized in FCS, resulted in an apparent K_D _of 9.16 μM (not shown). Nonspecific binding was more pronounced with serum-solubilized than with HP-γ-CD-solubilized curcuminoids. Since it can be assumed that the cellular binding affinity for curcuminoids *per se *remains constant, the variations in the apparent binding K_D_s may be interpreted as differences in affinities between curcuminoids and solubilization vehicles. However, the binding dose curves were generated with increasing amounts of pure solubilized curcuminoids and this does not reflect the situation *in vivo*, where the i.v. injected curcuminoids are immediately diluted with excess blood. The influence of serum dilution on cellular binding was, therefore, further examined to reflect this circumstance.

Curcuminoid preparations originally used for i.v. injections (24 mM and 6 mM HP-γ-CD-solubilized, 4 mM mouse serum-solubilized) were diluted 1:20 with DMEM or substituted with increasing proportions of mouse serum without curcuminoids. This resulted in total serum concentrations ranging from 0 to 95% for curcuminoids solubilized in HP-γ-CD and 5 to 100% for serum-solubilized curcuminoids. In all cases, cellular binding decreased with increasing serum concentrations in a manner consistent with a hyperbolic decline as described elsewhere [36] (Figure 8B). However, the decline in binding with increasing serum concentrations was dependent on the concentration of the curcuminoids in the media. Therefore, at maximal serum concentrations cellular binding remained at 39% (24 mM curcuminoids), 15% (6 mM) and 12% (4 mM) relative to the respective binding without added serum. These results are consistent with the notion that serum components, foremost albumin [29], act as competitors for cellular binding via the affinities of their interaction domains, which retain the curcuminoids in solution.

**Figure 9:**
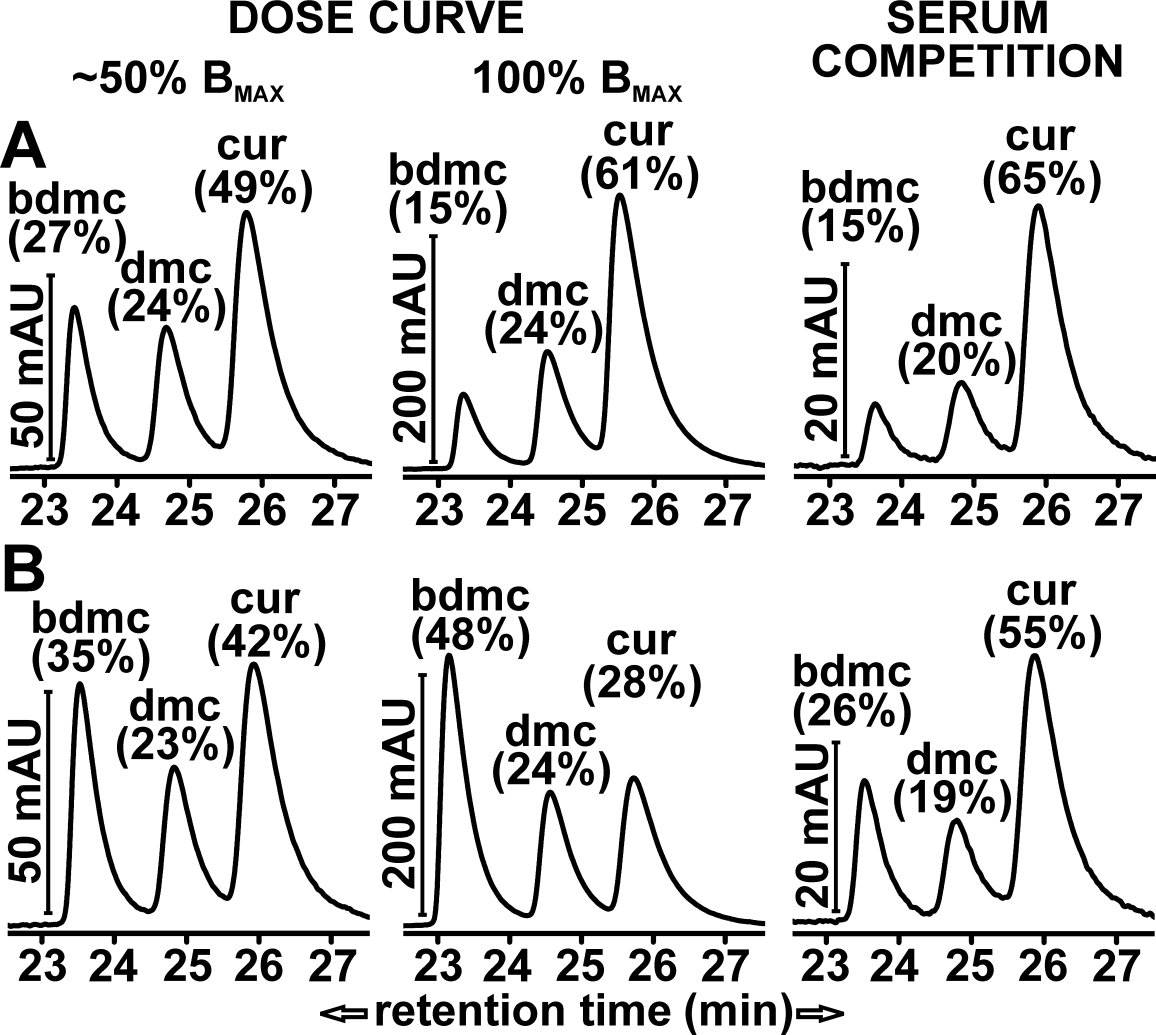
**Distribution of curcuminoids bound to NT2/D1 cells**. **(A) **Distribution of mouse serum-solubilized curcuminoids bound to NT2/D1 cells. Left panel: Chromatogram of cells incubated with curcuminoid concentrations near the K_D _value (approximately 50% B_MAX_) obtained from the dose curve shown in Figure 8A (O). Middle panel: Chromatogram of cells incubated at saturating curcuminoid concentrations (≥ B_MAX_, 200 μM curcuminoids added to medium at a total of 5% serum). Right panel: Same as middle panel except that cells were incubated with 100% serum. The middle and right panels illustrate the starting and end points of the serum competition curve shown in Figure 8B (O). The relative contribution of each curcuminoid is indicated as a percentage of the total. **(B) **Distribution of HP-γ-CD-solubilized curcuminoids bound to NT2/D1 cells. Left panel: Chromatogram of cells incubated with curcuminoid concentrations near the K_D _value (approximately 50% B_MAX_) obtained from the dose curve shown in Figure 8A (■). Middle panel: Chromatogram of cells incubated at saturating curcuminoid concentrations (≥ B_MAX_, 300 μM curcuminoids added to medium without serum). Right panel: Same as middle panel except that cells were incubated with 95% serum. The middle and right panels illustrate the starting and end points of the serum competition curve shown in Figure 8B (□).

Differences in cellular binding of individual curcuminoids were also observed, depending on whether they were solubilized in serum (Figure 9A) or HP-γ-CD (Figure 9B). At lower curcuminoid concentrations within the vicinity of the K_D _values (50% B_MAX_; Figure 9A, B, left panels), the distribution of cellular curcuminoids largely reflected the composition of the curcuminoids in the respective media (compare Figure 1A). However, under saturating conditions (≥ 100% B_MAX_; Figure 9A, B, middle panels), which represents the curcuminoid concentrations employed in the serum competition experiments (Figure 8B), the primary component bound to cells incubated with curcuminoids solubilized in serum was CUR (61%), whereas BDMC (48%) predominated in cells incubated with curcuminoids solubilized in HP-γ-CD. Indeed, both CUR and BDMC were the main contributors to nonspecific cellular binding during incubation with the respective solubilization vehicles. During serum competition of curcuminoids solubilized in serum, cellular binding decreased concordantly so that the relative distribution of bound curcuminoids remained unchanged (Figure 9A, right panel). In contrast, serum competition of curcuminoids solubilized in HP-γ-CD reduced cellular binding of BDMC more robustly so that the relative distribution of bound curcuminoids was shifted toward CUR (Figure 9B, right panel).

Overall, these results indicate that the relative affinity of curcuminoids for HP-γ-CD is weaker than for mouse serum components, which causes a reduction in the apparent cellular binding K_D_. Furthermore, the relative affinity of BDMC for HP-γ-CD is weaker than that of CUR, which causes this component to preferentially bind to cells at higher curcuminoid concentrations. The preferential serum competition of cellular binding of BDMC in media containing curcuminoids solubilized in HP-γ-CD is consistent with this idea. In general, the differences in cellular binding characteristics observed between curcuminoids solubilized in HP-γ-CD or serum are primarily noticed when these are used in their pure form. Upon dilution with serum and at lower curcuminoid concentrations, which are conditions rapidly established *in vivo*, these differences become minimized.

## Discussion

The oral administration of curcumin to humans, mice and rats has resulted in plasma levels typically not exceeding 1 μM concentrations and similarly low tissue levels (for recent reviews and references therein see: [26-28,44]). Alternatively, curcumin has been delivered to mice or rats by intraperitoneal (i.p) injection at dosages of 6 to 100 mg/kg body weight [15,45-47]. For this purpose, curcumin was either dissolved in DMSO [45-47] or in NaOH followed by neutralization [15]. In those studies, peak curcumin plasma concentrations were within the range of approximately 3.5 to 25 μM and brain levels approximately 1 to 2 nmol/g. After i.v. injection in rats of 10 mg/kg curcumin solubilized in a cocktail containing DMA/PEG/dextrose, initial plasma concentrations of approximately 27 μM were reported [46]. Similar results were reported in another study in which curcumin had been solubilized in glycerol formal and i.v. injected at a dosage of 40 mg/kg [48]. By comparison, in the present study mice were i.v. injected with 0.1 ml of 24 mM curcuminoids solubilized in 10% HP-γ-CD. This represents a total dose of 0.84 mg or approximately 33 mg/kg curcuminoids. Under these conditions, initial curcuminoid plasma concentrations of about 100 μM were attainable. Adjusted for the total dose applied, these concentrations are similar to those observed for the rat [46], but they resulted in transient brain concentrations of approximately 47 nmol/g (Figure 4), which were higher than those reported in any other study. Similarly, a four-fold higher curcuminoid dose (approximately 134 mg/kg) administered by s.c. injection yielded maximal plasma concentrations of approximately 23 μM and brain levels of approximately 8 nmol/g. Although these amounts are lower than those achieved by i.v. injection, the parental curcuminoids were released gradually from the injection site and they persisted longer in both plasma and brain (Figure 5). These levels are also higher than those typically observed after i.p. injection (see above). However, in one study curcumin was administered at a much lower dose (3 mg/kg) and this yielded relatively high brain levels of approximately 3.2 nmol/g at four hours after intramuscular injection [15].

The injection protocol presented here combines the high solubility of curcuminoids in 10% HP-γ-CD with the relatively low level of toxicity of the carrier vehicle. In addition, HP-γ-CD does not cause apparent immune reactions that are associated with the use of heterologous serum to solubilize curcuminoids. However, at the highest doses used for i.v. injection (33 mg/kg), toxic reactions do develop and these may represent the maximum amount of curcuminoids tolerated before lethal effects occur. After a single i.v. injection, high plasma levels are exceedingly transient and the concentration of native curcuminoids drops to insignificant levels within 20 minutes (Figure 4). The decline in the level of circulating curcuminoids can be considered due to rapid metabolism combined with widespread binding/uptake to cells and tissues.

The binding of curcuminoids to cells in culture has been described in detail elsewhere [36]. Those studies were expanded to include preparations employed here, where curcuminoids had been sequentially solubilized in mouse serum and 10% HP-γ-CD (Figure 8). This was done by first adding curcuminoids as a solid powder followed by the addition of DMSO-dissolved curcuminoids. This method results in a maximal solubility of curcuminoids and a relatively balanced composition [29]. The apparent binding K_D_s in these preparations were higher for curcuminoids solubilized in mouse serum (14.88 μM) than in FCS (9.16 μM). Since mouse serum has the capacity to solubilize higher concentrations of curcuminoids than FCS (3 to 4 mM vs. approximately 1.7 mM [29]), it is likely that these differences in binding K_D_s reflect differences in affinities or concentrations between the curcuminoid interaction domains in serum components, as it may be reasonably assumed that the cellular binding affinities remain constant. It is further expected that curcuminoids solubilized in sera from different species or possibly different preparations from the same species, will produce different apparent cellular binding K_D_s due to their variable serum compositions. In addition, alternative solubilization vehicles may have differential affinities for individual curcuminoids. This is exemplified by the cellular binding of individual curcuminoids solubilized in HP-γ-CD (Figure 9). Although curcuminoids solubilized in either HP-γ-CD or serum have similar compositions (Figure 1), the primary curcuminoids bound to cells at saturating concentrations were BDMC and CUR, respectively. However, upon dilution with excess serum, both preparations converged to the same compositions of cellular-bound curcuminoids (Figure 9). Although these observations were made with cultured cells, it is likely that similar binding occurs *in vivo*. Indeed, the extensive binding of curcuminoids to brain (Figure 6) and other organs (not shown) shows a similar distribution pattern as that obtained with cultured cells incubated with curcuminoids and excess serum. Except for considerations relating to solubilization capacity, toxicity or immune reactions, it is in this case irrelevant whether curcuminoids are solubilized in serum or HP-γ-CD.

The metabolic conversion of curcuminoids after i.v. injection is rapid and it includes both conjugation and reduction products (Figure 4). Since conjugation takes place in the liver, intestines and kidneys, the resulting products are observed primarily in plasma but also at the sites of excretion [48-51]. In addition to conjugation products, the hexa- and octahydrocurcuminoid reduction products are also prominently represented in the circulation. It is likely that these are primarily contributed by curcuminoids that were taken up by peripheral cells and tissues, metabolized locally, and subsequently released into the bloodstream. In contrast, after s.c. injection the curcuminoid reduction products are not readily detected in plasma. This is most likely due to the more gradual release from tissues followed by rapid excretion. Although only the monoconjugates of sulfate and glucuronide were investigated here, it is likely that mixed diconjugates are also produced [48,50,51]. Some studies have reported the formation of tetrahydrocurcumin in mice [47,49], which was not detected in this study. Instead, the hexa- and octahydrocurcuminoids predominated. In addition, different reduction products with different physical characteristics, here referred to as dihydrocurcuminoidols, were detected in the brain. These compounds were first identified as the final reduction products in the teratocarcinoma cell line NT2/D1 [36], while its presence *in vivo *has not been previously reported. However, dihydrocurcuminoidols are most prominently produced after perfusion under post-mortem conditions. Their time-dependent production in the brain following i.v. injection also seems to be correlated with the amount of unmodified curcuminoids present. It can, therefore, not be excluded that this is a post-mortem effect that rapidly occurs during the removal of tissues. The enzyme systems responsible for the reductive conversion of curcuminoids have not yet been conclusively identified. However, it appears that octahydrocurcuminoids are sequentially generated from hexahydrocurcuminoids. For example, during perfusion only hexahydrocurcuminoids together with smaller amounts of dihydrocurcuminoidols were generated and since the post-mortem conversion to octahydrocurcuminoids was blocked, there was a larger relative accumulation of hexahydro-BDMC compared to *in vivo *conditions (Figure 6). This indicates that the overall conversion to octahydrocurcuminoids requires distinct enzyme systems with different substrate specificities and metabolic requirements. The more efficient generation of octahydro-BDMC than octahydro-CUR is consistent with this notion, as is the gender-specific generation of reduction products in the rat [39]. The curcumin reducing enzymes have also been found to be distributed between cytoplasmic and microsomal compartments [38]. In addition, different cell lines in culture produce different reduction products. For example, in the astrocytoma cell line CCF-STTG1, reduction proceeds to the octahydrocurcuminoid stage, whereas in HeLa cells hexahydrocurcuminoids are the end products [36]. These cell lines also produce varying amounts of dihydrocurcuminoidols [36]. Based on these observations, possible alternative reduction pathways for curcuminoid reduction are proposed (Figure 7).

## Conclusions

This study was undertaken to examine the feasibility of using an injectable form of curcuminoids to modulate the formation of amyloid plaques in brains of Alzheimer transgenic mice. This was based on the premise that relatively low concentrations of curcumin suffice to eliminate or reverse amyloid fibril formation *in vitro*. Furthermore, a number of studies have reported a reduction in plaque load following long-term (four to nine months) oral uptake of curcumin in similar transgenic mouse models. Despite low plasma levels, the plaque load was reduced to 48 to 69% of control values [9,15-17]. However, including curcumin in the diet represents chronic exposure to low levels of curcumin. In this study, the amount of curcuminoids injected was relatively high, yielding a significant rapid distribution into both plasma and tissues. Nevertheless, the high rate of metabolism rendered these levels short-lived. In effect, the long-term intermittent exposure at intervals of once/week had no discernable influence on plaque formation, although at four-fold higher dosage and with a frequency of twice/week for one month, a reduction in plaque load to about 70% of control values was observed. Indeed, similar plaque load reductions were reported in the same strain of mice after daily tail vein injections for seven days using only 7.5 mg/kg curcumin [8]. It is, therefore, conceivable that inhibition of plaque formation and plaque resolution depends on a more frequent administration of curcuminoids. Since frequent long-term tail vein injections in mice are problematic, these could be augmented or substituted with better tolerated subcutaneous injections in future studies. These modes of administration could also be complemented with formulations for improved oral uptake. For example, preliminary studies with HP-γ-CD-solubilized curcuminoids in drinking water yielded consistent plasma concentrations in the range of 0.5 to 1 μM (not shown). Therefore, combining the oral uptake of curcuminoids via both food and drinking water with injection protocols may result in more effective procedures for plaque prevention and elimination.

## Abbreviations

AD: Alzheimer's disease; APP: amyloid protein precursor; BDMC: bisdemethoxycurcumin; CUR: curcumin; DMA, *N*: *N*-dimethylacetamide; DMC: demethoxycurcumin; DMEM: Dulbecco's Minimal Essential Medium; DMSO: dimethyl sulfoxide; FCS: fetal calf serum; HP-γ-CD: 2-hydroxypropyl-γ-cyclodextrin; i.v.: intravenous; PBS: phosphate-buffered saline; PEG: polyethylene glycol; s.c.: subcutaneous

## Competing interests

The authors declare that they have no competing interests.

## Authors' contributions

All authors contributed to the manuscript. WQ devised the protocols and performed the biochemical experiments. NS and JR carried out all animal related procedures. All authors read and approved the final manuscript.
